# Reasons for Preference of Home Delivery with Traditional Birth Attendants (TBAs) in Rural Bangladesh: A Qualitative Exploration

**DOI:** 10.1371/journal.pone.0146161

**Published:** 2016-01-05

**Authors:** Bidhan Krishna Sarker, Musfikur Rahman, Tawhidur Rahman, Jahangir Hossain, Laura Reichenbach, Dipak Kumar Mitra

**Affiliations:** 1 Centre for Reproductive Health, International Centre for Diarrhoeal Disease Research, Bangladesh (icddr,b), GPO Box-1000, Dhaka, Bangladesh; 2 Care Bangladesh, Pragati Insurance Bhabhan, 20–21 Kawran Bazar, Level 9–13, Dhaka, 1215, Bangladesh; 3 Senior Associate and Deputy Director for Research, The Evidence Project, Population Council, Washington, District of Columbia, United States of America; 4 Former Director and Social Scientist, Centre for Reproductive Health, International Centre for Diarrhoeal Disease Research, Bangladesh (icddr,b), GPO Box-1000, Dhaka, Bangladesh; 5 International Center for Maternal and Newborn Health, Department of International Health, Johns Hopkins Bloomberg School of Public Health, Johns Hopkins University, Baltimore, Maryland, United States of America; London School of Economics, UNITED KINGDOM

## Abstract

**Background and Objectives:**

Although Bangladesh has made significant progress in reducing maternal and child mortality in the last decade, childbirth assisted by skilled attendants has not increased as much as expected. An objective of the Bangladesh National Strategy for Maternal Health 2014–2024 is to reduce maternal mortality to 50/100,000 live births. It also aims to increase deliveries with skilled birth attendants to more than 80% which remains a great challenge, especially in rural areas. This study explores the underlying factors for the major reliance on home delivery with Traditional Birth Attendants (TBA) in rural areas of Bangladesh.

**Methods:**

This was a qualitative cross-sectional study. Data were collected between December 2012 and February 2013 in Sunamganj district of Sylhet division and data collection methods included key informant interviews (KII) with stakeholders; formal and informal health service providers and health managers; and in-depth interviews (IDI) with community women to capture a range of information. Key questions were asked of all the study participants to explore the question of why women and their families prefer home delivery by TBA and to identify the factors associated with this practice in the local community.

**Results:**

The study shows that home delivery by TBAs remain the first preference for pregnant women. Poverty is the most frequently cited reason for preferring home delivery with a TBA. Other major reasons include; traditional views, religious fallacy, poor road conditions, limited access of women to decision making in the family, lack of transportation to reach the nearest health facility. Apart from these, community people also prefer home delivery due to lack of knowledge and awareness about service delivery points, fear of increased chance of having a caesarean delivery at hospital, and lack of female doctors in the health care facilities.

**Conclusions:**

The study findings provide us a better understanding of the reasons for preference for home delivery with TBA among this population. These identified factors can inform policy makers and program implementers to adopt socially and culturally appropriate interventions that can improve deliveries with skilled attendants and thus contribute to the reduction of maternal and neonatal mortality and morbidity in rural Bangladesh.

## Introduction

Bangladesh has made remarkable progress towards achieving Millennium Development Goals (MDG) 4 and 5. According to the Bangladesh Maternal Mortality Survey (BMMS-2010), MMR declined from 322 in 2001 to 194 per 100,000 live births in 2010, a remarkable 40 percent decline in 9 years. The average rate of decline from the base year has been about 3.3 percent per year, compared to the 3 percent average annual rate of reduction required for achieving Millennium Development Goal 5 (MDG-5) in 2015 [1]. The Government of Bangladesh is committed to achieving MDG-5 by reducing the maternal mortality ratio (MMR) to 143 deaths per 100,000 live births by 2015 and increasing skilled attendance at birth to 50 percent by 2016 [2]. But achieving the MDG-5 target of 50% skilled attendant by 2015 remains a great challenge, especially in rural areas of Bangladesh. The majority of deliveries still take place at home (62%), and more than 56% deliveries are assisted by traditional birth attendants (TBAs) or relatives while medically trained personnel conduct only 42% of all births, both at home and in facilities at the national level. The deliveries which take place at home and are assisted by TBAs are often performed in unsafe and unhygienic conditions resulting in increased risk of maternal and child morbidity and mortality. In Bangladesh, 36% women do not receive any antenatal care from medically trained persons and the situation is much worse in rural areas (41%) than the urban area (21%) [3]. Therefore, concerted efforts are required to overcome persistent geographic and socio-economic inequities and cultural and normative barriers that impede maternal health service utilization, thus contributing to maternal deaths and morbidities.

This low maternal health service utilization is also affected by a shortage of health work force—especially in rural areas. Bangladesh is one of the countries identified as having a severe shortage of health workers. Bangladesh has approximately five physicians and two nurses per 10,000 population and the ratio of nurses to physicians is only 0.4. There are approximately 12 unqualified village doctors and 11 sales people at drug retail outlets per 10,000 populations with the latter being uniformly spread across the country [4]. In addition to limited health care facilities and qualified health professionals, social norms and taboos lead to practices that are potentially harmful and contribute to maternal and child mortality. These norms and practices have been documented in both rural and urban areas. Several studies have identified a variety of cultural norms and superstitions that still exist in Bangladesh and are harmful for achieving healthy and safe motherhood. Many of these practices involve restriction on women’s mobility and the consumption of inadequate food during pregnancy and the postnatal period. For example one common belief is that eating more food during pregnancy can lead to having large babies that may cause obstructed labor and thus require a caesarean delivery [5]. Some common harmful practices during pregnancy and childbirth include: internal manipulation and massage, introduction of oils into vagina, use of tight abdominal bands during labor, pulling the umbilical cord, choking or inducing vomiting in the woman to expedite placental delivery, etc [6]. The BDHS-2014 data shows that 64% women receive ANC from medically trained providers and women who did not seek ANC thought that the check-up was not needed [3]. Studies have also emphasized the socio-cultural barriers that can limit women’s use of professional care regardless of quality of care in the facility [7–8].

The improvement of the maternal and child health situation in Bangladesh is not uniform across the country. For example, there is significant regional variation in MMR; the MMR in Khulna Division is only 64/100,000 live births compared to an MMR of 425/100,000 live births in Sylhet Division. Apart from the regional variation, MMR also varies by rural and urban areas (MMR in rural areas is almost 200/100,000 live births compared to 178/100,000 live births in urban areas) [1]. Recent BDHS-2014 data also shows poor utilization of healthcare services and low antenatal care coverage and poor rates of facility delivery in Sylhet Division [3]. This study was conducted in the Sunamganj district of Sylhet Division; the area which shows the lowest maternal and child health indicators in Bangladesh [9]. Sunamganj, located in the north eastern part of Bangladesh, is one of the remotest areas of the country. This region is surrounded by *Haors (*locally called). *Haor* is a wet land ecosystem with a bowl or saucer shaped shallow depression also known as a back swamp area. The economic situation of this area is not well off. As per the Bangladesh Bureau of Statistics (BBS-2010), 26% population in Sunamganj are poor (belongs to upper poverty line), of which 21% population are extremely poor (belongs to lower poverty line) [9]. A baseline survey conducted in Sunamganj district in 2012-13showed that almost 74% of households had an average monthly income less than 10,000 BDT (147$) [10]. The objectives of the study were to explore the reasons why community women prefer home delivery with the help of TBA and to identify the social and cultural barriers that deter women from seeking delivery care from medically trained providers in facilities.

## Materials and Methods

### Ethics Statement

This study was approved by the Research Review Committee (RRC) and Ethical Review Committee (ERC) of icddr,b as part of Institutional Review Board (IRB) approval prior to the start of data collection.

### Study design and methods of data collection

This was a qualitative cross-sectional study which applied several qualitative methods to address the research objectives. Data collection methods included key informant interviews (KII) with stakeholders, formal and informal health service providers and health managers and in-depth interviews (IDI) with community women to capture the range of information and perspectives needed to fully explore the reasons that hinder rural community women from accessing skilled birth attendants during child birth.

### Study site

We conducted this study in Sunamganj district which is full of *haors* and *baors* (marshy areas). Sunamganj district is one of the most remote and hard-to-reach areas of Bangladesh. The district consists of 11 Upazilas with 87 unions. According to the Population and Housing Census-2011 data, the total population of this district is 2,467,968, density of population is 659 per square km, and the average household size is 5.58. The literacy rate of this district is 35%. In this district, 87% of the population is Muslim, 13% Hindu, 0.1% Christian and 0.002% Buddhist (BBS, 2013) [11]. Most of the people in study areas are involved with agricultural activities. We conducted this study in three sub-districts of Sunamgonj district. Most of the roads in the study areas are made of mud, some of which are broken. The entire study area remains under water for half of the year. It is very difficult to move from one place to another in the villages during the rainy season; villagers have to use bamboo bridges to cross the *haors* or marshy areas. During the rainy season, the only form of transportation is boat, without which one cannot move from one place to another. However, boats are not always available. After the rainy season is over, villagers mostly move from one place to another on foot. The only vehicle used is the motorbike which pregnant women cannot easily use, especially as the road remains dilapidated long after the rainy season is over. Therefore, poor road conditions and lack of availability of transportation in both the rainy and dry seasons deter women from seeking facility based maternal and child health care in this study area. Residents of the study area usually seek health services from informal providers such as: village doctors or drug sellers in and around the community.

### Study participants and sampling procedure

**Fig 1:**
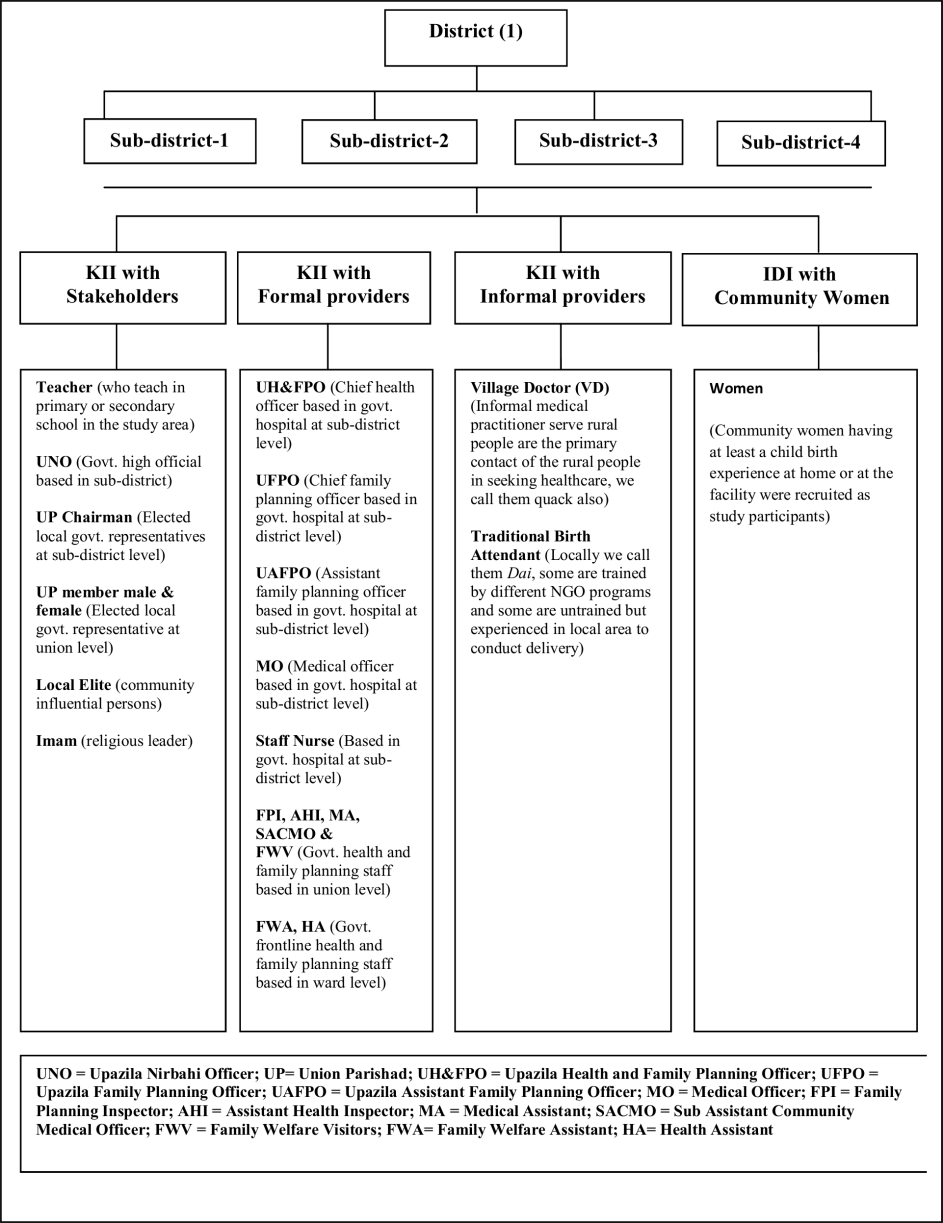
Detailed sampling frame.

We included several categories of study participants such as stakeholders which included influential community members, formal and informal maternal and child health (MCH) service providers, as well as community women. Stakeholders included Upazila Nirbahi Officers (UNO), Upazila Chairman, and Union Parisad (UP) chairman, UP member, as well as community influential persons such as Imamas (Muslim religious leader) and teachers. As part of the formal MCH service providers we interviewed government Family Welfare Assistants (FWAs), Family Welfare Visitors (FWVs), Health Assistants (HAs), Upazila Family Planning Officers (UFPOs), Upazila Health & Family Planning Officers (UH&FPOs), obstetrics and gynecology consultants and nurses. We interviewed Village Doctors (VDs) and Traditional Birth Attendants (TBAs) as informal MCH service providers. Finally, we interviewed community women with at least one living child below one year of age at the time of interviews to get their own experiences and perceptions regarding home delivery and preferences for TBAs. We applied a snowball sampling technique to identify the eligible respondents for both KIIs and IDIs. This snowball sampling approach helped us to easily identify eligible respondents. Those TBAs and VDs who were most popular or who conducted or assisted with high volume delivery in the last one year were identified and selected as study participants and identified based on discussions with the local community. While qualitative study participants were purposively selected in the field, several eligibility criteria were used as a sampling frame including: education for the women (<5 vs. >5 class); years of involvement as community leader for the local elite e.g., UP member, Chairman, Imam, Teacher, etc; job duration for the formal and informal health service providers (experience for 5 years or more) in the maternal and child health and Family Planning field. We used purposive sampling to select the respondents in consultation with the community people living in the study area who could provide the most appropriate information to serve the purpose of the study. We shared our study objectives among the community people and asked them to let us know who would be best person to discuss on this issue. Therefore, community people themselves identified the key informants and based on our selection criteria we went to respective informants to conduct interviews. A detailed sampling frame is presented in Fig 1.

### Sample Size

We conducted 21 KIIs with stakeholders, 21 KIIs with formal health service providers, and 12 KIIs with informal providers at the community and facility level. In addition, 12 IDIs with community women were conducted to explore their perceptions regarding MNCH care seeking behavior as well as preference for TBA and home delivery. A total of 66 interviews were administered. These numbers were determined in the field based on data saturation derived from concomitant data analysis. We prepared a matrix where we used to input data regularly to identify data saturation level. After the data saturation level was found, we stopped doing interviews. Sample size details are provided below in Table 1.

**Table 1:** Summary of sample size.

Study participants	Sample size
KII with stakeholders	21
KII with formal health service provider	21
KII with informal health service provider (Village Doctor)	6
KII with informal health service provider (TBA)	6
IDI with community women having one year child	12
Total	66

### Data collection, tools and quality checking

Data were collected between December 2012 and February 2013. A team of experienced researchers with social science background and training in qualitative research methods were responsible for the data collection. Separate guidelines were prepared and pre-tested to conduct KIIs and IDIs with different study participants. It took about one hour to complete each KII and IDI. The interviews took place in the home of the participants or at a suitable place of their choice; interviewers tried to secure a setting with the fewest disturbances. Full informed consent was taken from each respondent prior to the interview. Interviewers of this study clearly conveyed to the respondents the objectives of the study, why we invited them to participate in the study, their role in it, risk and benefits, privacy, anonymity and confidentiality, right not to participate and withdraw etc. After getting acquainted with all of these issues, the respondents and only the respondents decided whether they would participate in this study or not. We also made clear that they could stop at any time without any obligation during interview. We also obtained their consent to audio record the interview. We used ID numbers for each respondent to ensure their identities remained anonymous. However, the privacy, anonymity and confidentiality of information were maintained in such a way that participants in no way could be identified. Literate participants read the consent paper first and then gave their consents signing the consent form. Those who could not sign gave left thumb impression. A signature or left thumb impression was also obtained from a witness during the consent process. The interviewers ensured that that no one would be present with the participant during the interview to enable uninterrupted collection of in-depth information. Data collectors were responsible for transcribing, coding and summarizing the interviews.

Field Research Supervisors closely monitored the data collection team and communicated regularly with the study investigators. The nature of the field site was very remote and the dialect of this area was different from other parts of the country. Study investigators made frequent field visits to monitor data collection and provide necessary feedback on an urgent basis. We established a strong mobile phone network between all data collectors, field supervisors and study investigators to monitor the study progress and to resolve any important issues arising in the field. Standardization procedures of data collection were done before and during the study to ensure similarity in measurement techniques among staff members, and standard study protocols were established for all data collection procedures. Two separate guidelines were used for data collection and then were translated into the languages appropriate to the communities. The adequacy of these translations was checked by one of the study investigators.

### Data analysis

We prepared an outline of data analysis based on the study objectives. We engaged researchers with a qualitative background and experience in data analysis using a framework approach. The framework method is a systematic and flexible approach to analyze qualitative data and is appropriate for use in this type of research setting. The framework creates a new data structure that is helpful to summarize the data in a way that can support answering the research questions [12]. While conducting interviews, we also took notes. All the field notes and interviews (with or without audio recording) were considered for the data analysis process and transcribed on the same day. We did verbatim transcriptions immediately after recording. The data were systematically indexed, synthesized and interpreted to provide explanations for the findings. Results from respondents from different areas were compared to strengthen the validity of the findings. Researchers involved in the analysis developed a set of codes that was used to manage and organize the data that were analyzed manually.

## Results

The results are presented in broad themes and within each theme several sub-themes emerged. We explored why women prefer home delivery and the reasons behind their preference for conducting deliveries with a TBA. The results are presented based on the frequency of reasons mentioned. We also described the characteristics of the study participants. The main questions asked to the study participants are presented in Table 2.

**Table 2:** Key questions asked to study participants.

Study participants	Key questions asked
**Women**	
	Where do you usually go to seek maternal health services and why?
	Where do you prefer to have your delivery or child birth and why?
	Whom do you prefer to conduct your delivery and why?
	If you prefer informal providers like TBA, then, why? In your opinion, what are the reasons that lead you to receive delivery care from TBA?
	If you prefer other than TBA, then why?
**Formal providers and Village Doctors (Informal provider)**	
	What are the specific health services that you provide to pregnant women in this area?
	From your observation, where do most pregnant women usually go to conduct delivery?
	If you think that most women prefer home delivery then what could be the possible reasons behind it?
	If you think that most women prefer facility delivery then what could be the possible reasons behind it? And if they do not prefer facility delivery, why not?
	In your opinion, why do women conduct delivery at home by TBA? Why do not they conduct delivery from skilled or qualified provider?
**TBA (Informal provider)**	
	What are the specific health services that you provide to pregnant women in this area?
	From your observation, where do most pregnant women usually go to conduct delivery?
	If you think that most women prefer home delivery then what could be the possible reasons behind it?
	If you think that most women prefer facility delivery then what could be the possible reasons behind it? And if they do not prefer facility delivery, why not?
	In your opinion, why do women conduct delivery at home by you? Why do not they conduct delivery at health facility?
**Stakeholders**	
	From your observation, where do most of the pregnant women usually go to conduct delivery in your area?
	If you think that most women prefer home delivery then what could be the possible reasons behind it?
	If you think that most women prefer facility delivery then what could be the possible reasons behind it? And if not prefer then why don’t they prefer facility delivery?
	In your opinion, why do women conduct delivery at home by TBA? Why do not they conduct delivery from skilled or qualified provider?

### Characteristics of study participants

#### KII participants

Majority (83%) of study participants were aged 30 years and above. Among the participants 63% were male and 37% were female. Sixty five percent participants were Muslims and the rest of the participants were Hindus by religion. Majority (69%) of the participants had an education of class 10 and above and 22% had an education from class 5 to 9. Fifty six percent of participants had a monthly household income of more than ten thousand taka while 30% had a monthly household income of less than five thousand taka. We included participants from different occupations such as: service (48%), agriculture (11%), village doctor (11%), TBA (11%) and housewife (6%). Table 3 presents the socio-demographic characteristics of KII participants.

**Table 3:** Socio-demographic characteristics of the KII participants.

Socio-demographic traits	KII (Influential Person)	KII (Informal Provider)	KII (Formal Provider)	Total KII participants
**Age**				
Below 30 years	1(5%)	3(25%)	5(24%)	9(17%)
30 or above	20(95%)	9(75%)	16(76%)	45(83%)
Total	21(100%)	12(100%)	21(100%)	54(100%)
**Sex**				
Male	17(81%)	6(50%)	11(52%)	34(63%)
Female	4(19%)	6(50%)	10(48%)	20(37%)
Total	21(100%)	12(100%)	21(100%)	54(100%)
**Religion**				
Islam	16(76%)	6(50%)	13(62%)	35(65%)
Hinduism	5(24%)	6(50%)	8(38%)	19(35%)
Total	21(100%)	12(100%)	21(100%)	54(100%)
**Respondent’s level of education**				
No formal education	0(0%)	5(42%)	0(0%)	5(9%)
Class 1 to Class 4	0(0%)	0(0%)	0(0%)	0(0%)
Class 5 to Class 9	11(52%)	1(8%)	0(0%)	12(22%)
Class 10 and above	10(48%)	6(50%)	21(100%)	37(69%)
Total	21(100%)	12(100%)	21(100%)	54(100%)
**Respondents Occupation**				
Agriculture	6(29%	0(0%)	0(0%)	6(11%)
Business	4(19%)	0(0%)	0(0%)	4(7%)
Housewife	3(14%)	0(0%)	0(0%)	3(6%)
Imam	3(14%)	0(0%)	0(0%)	3(6%)
Service	5(24%)	0(0%)	21(100%)	26(48%)
Village doctor	0(0%)	6(50%)	0(0%)	6(11%)
TBA	0(0%)	6(50%)	0(0%)	6(11%)
Total	21(100%)	12(100%)	21(100%)	54(100%)
**Monthly household income of respondent (BDT)**				
Below 5000	10(48%)	6(50%)	0(0%)	16(30%)
5001–10000	3(14%)	3(25%)	2(9.5%)	8(14%)
10001 to above	8(38%)	3(25%)	19(90.5%)	30(56%)
Total	21(100%)	12(100%)	21(100%)	54(100%)

#### IDI participants

All the IDI participants were females whose occupation was housewife. Most of the participants (10 out of 12) were aged below thirty years. Fifty eight percent were Muslims and the rest were Hindus by religion. Half (6 out of 12) of the participants had education ranging from class1 to 5 with the remainder having education from class 6 to 8 (25%) and having no formal education (25%). Half of the participants had monthly income below five thousand taka (BDT). Table 4 presents socio-demographic characteristics of IDI participants.

**Table 4:** Socio-demographic characteristics of IDI participants.

Socio-demographic traits	IDI (Women)
**Age**	
Below 30 years	10(83%)
30 or above	2(17%)
Total	12(100%)
**Religion**	
Islam	7(58%)
Hinduism	5(42%)
Total	12(100%)
**Respondent’s level of education**	
No formal education	3(25%)
Class 1 to Class 5	6(50%)
Class 6 to Class 8	3(25%)
Total	12(100%)
**Respondents Occupation**	
Housewife	12(100%)
Total	12(100%)
**Monthly household income of respondent (BDT)**	
Below 5000	6(50%)
5001–10000	4(33%)
10001 to above	2(17%)
Total	12(100%)

### Why people prefer home delivery with a TBA

Several common themes emerged from the data analysis that provides insights into why women in this rural and hard to reach area prefer delivery at home and with a TBA. These themes are described below.

#### Traditional views prevail

Traditionally all the TBAs are women and delivery by TBA is an established cultural practice in Bangladesh. The study area is a conservative place with a Muslim majority where the education of women is poor. Elderly women who make decision within the family have good relations with TBAs in the community. One-third of the women interviewed and a few stakeholders mentioned that giving birth at home was considered a tradition. This was especially true among older respondents. Often the mother-in-law prohibits her daughter-in-law from going to a hospital because she herself did not go to a hospital for delivery. The importance of the views of elders, including mothers-in-law remain an important factor of influence in decision making around delivery. The following quote from one of the stakeholders illustrates this underlying view.

***“In the past, women used to have their deliveries at home; and all deliveries were successful with one or two exceptions.…, so, why do women need to go to hospital to have their delivery?”*** (Age-67, Sex-Male, Education-8 class, Occupation-Agriculture)

A woman respondent in the above mentioned context quoted

***“Giving birth at home is good*. *If delivery happens at home*, *people would not be able to know about it*, *by the grace of Allah delivery happened successfully which is good as well*, *so*, *there is widely prevalent concept that I would rather die at home than go to hospital for delivery”*** (Age-23, Sex-Female, Education-class 5, Occupation- Housewife)

The village doctors (5 out of 6) and TBAs (2 out of 6) opined that if a delivery is conducted outside the home, the prestige and social status of the family is downgraded in the community. In this regard, one TBA quoted,

***“In this community*, *people think that*, *if a woman goes to hospital for delivery then women have to be nude in front of un-known male doctor*. *Besides*, *women also think that*, *if they go to hospital for delivery*, *their delivery will happen by caesarean instead of normal delivery*, *community people will not take it simply rather they will insult women*. *This issue hampers their prestige”*** (Age-70, Sex-Female, Education-No formal education)

An important related issue that was raised focused on the social constraints of obtaining treatment from male doctors. Several respondents mentioned the fact that most of the doctors at the Upazila and district level hospitals are men. Four formal and five informal health care providers, two stakeholders and one woman said that women are ashamed of taking treatment from male doctors because they think that their social status and prestige will be diminished as a result. For example, a woman has the chance to have her reproductive organs exposed to a male provider which is a major barrier. In addition, women are less likely to discuss their problems or symptoms with a male doctor. Interestingly, the study participants who mentioned that women feel hesitant and shy to take maternity care from male doctors were all Muslims.

***“Women easily understand another woman’s voice; they cannot share their all problems with a male person or to a male doctor*, *as they feel shy with them*. *If delivery is conducted at home by TBA*, *women are free from any anxiety and they feel peace in their mind*.*”*** (Age-34, Sex-Female, Education-Class 10, Occupation-Teacher)

#### Poverty

The issue of inability to pay for a facility-based delivery was a consistently cited reason by all of the respondents for preferring home deliveries. All of the six village doctors, one-third of the TBAs, all of the women and one-third of the stakeholders interviewed said that most of the people in their area are poor and simply do not have enough money to pay for a facility-based delivery. Therefore, families prefer to deliver at home with the help of a TBA due to the lower price for a TBA and the option to compensate her with non-monetary items such as a *Shari* (a traditional dress worn by Bangladeshi women). A few respondents also mentioned that even better off people in their area may not be able to afford the high cost of delivery at hospitals or clinics. In this regard one of the respondents said,

***“It is good to have delivery at home*, *because it costs less at home but costs much higher at other places outside the home*. *We do not have to pay money to Dai (TBA) for her service*, *only a new Shari is enough to make her happy*.*”*** (Age: 62 years, Sex: Female, Education: Class five, Occupation -Elected Female UP Member)

Another respondent quoted,

“***TBA costs very small amount of money*, *and sometimes she even provides service free of cost”*** (Age: 22 years; Sex: Female; Education: Class 5; Occupation: Housewife)

#### TBA is considered a trusted and experienced provider

Thirteen formal providers, more than one-third of the stakeholders, and one-half of the TBAs interviewed gave the opinion that people consider TBAs to have a lot of experience in conducting deliveries and that is why they demand the services of TBAs. Women have also expressed their trust in the experience and skills of TBAs. Regarding this, a woman quoted

***“TBA has some special skills that doctors do not have and TBA cannot do what doctors can do*, *it takes long time for doctors to come to patient’s house*, *the situation of mothers deteriorates by this time*. *Besides*, *why we should go for doctors*, *TBA is much better than doctors”***. (Age: 18 years, Sex: female, Education: class four, Occupation: Housewife)

One Imam (religious leader) provided a similar opinion, he quoted

***“Village people conduct delivery with the help of TBA as they have faith in TBA; TBA is also good at conducting delivery*.*”***(Age: 34 years, Sex: Male, Education: Above class 10, Occupation-Imam)

All twelve women interviewed said that they usually give birth at home with TBAs because they help to give birth naturally and in familiar surroundings with known faces, which is a source of comfort for the women. The TBAs’ availability and her familiarity with the community were additional reasons shared by women for preferring their assistance during delivery.

Another respondent quoted,

**“*TBAs are local inhabitants and familiar to us for a long time*. *People think that old TBAs are skilled*, *they provide good services*. *Many people hire TBAs from neighboring villages*, *who have lot of experiences in assisting many deliveries without facing any difficulties*. *Women also go to those TBAs who conducted their previous deliveries*.*”*** (Age: 52 years; Sex: Male; Education: Above class 10; Occupation: Agriculture & Politics)

Another respondent quoted,

***“People think that TBAs are trained and have been conducting deliveries for a long time*. *TBAs can handle delivery cases very well*. *Giving birth at home gives mental peace*. *Only a woman can understand another woman’s problems very well*, *a woman cannot talk about all their problems with a man*, *she feels shame and does not feel fear while giving birth at home with the help of TBA; this is her mental peace*.*” (***Age: 34 years; Sex: Female; Education: Passed class 10; Occupation: Teacher)

#### Illiteracy and lack of knowledge

Seven formal providers and four stakeholders said that most of the people in the area are illiterate and do not have appropriate knowledge regarding maternal health services leading to home delivery with the help of TBA.

Usually community people do not want to go to a facility for delivery. They do not have the right information about the appropriate place for delivery as well as demerits of home deliveries. Rather, unless or until they are faced with severe complications with pregnant women, they do not feel to go to the facility. One of respondents quoted,

“***We go to TBA and prefer to give birth at home*. *We go to hospital only when complications occur*. *To me*, *home is good place to give birth”*** (Age: 22 years; Sex: Female; Education: Class 5; Occupation: Housewife)

A local leader shared his thoughts that the community people especially who have little education and awareness about the modern medical system prefer home delivery by TBA. In this regard, he quoted,

“***Here most of the deliveries take place at home by TBA because of illiteracy and lack of knowledge of the community people about facility delivery*. *So*, *the delivery complications become aggravated when TBA cannot manage it*. *Despite putting lot of efforts*, *when TBAs fail to conduct delivery then they invite village doctors to try*. *Even the village doctors cannot do it as they also do not have any special training*. *And if pregnant women are taken to hospital with this severe condition*, *it hardly brings any success*. *Sometimes*, *women die as a result”*** (Age: 46 years; Sex: Male; Education: Above class 10 & Diploma; Occupation: Business person cum local leader)

In terms of education of women, we did not notice any marked differences in the preference of home delivery by TBA among women of different educational levels.

#### Religious beliefs

Almost all the village doctors and one-seventh of the stakeholders opined that their village people believe in religious restrictions, conservativeness and strict rules of maintaining the Islamic principle of *purdah* that actually limit the mobility of women significantly. As previously mentioned, because this is a particularly religious and conservative area of Bangladesh, residents of Sunamganj believe that it is a sin to deliver outside of home because it violates *purdah*. One informal provider said

***“The women should conduct her delivery at home; if the delivery is conducted outside home it will be anti-Islamic”*** (Age-28, Sex-Male, Education-Class 10, Occupation-Village Doctor).

In this regard, a stakeholder quotes

***“We are Muslim*, *if male people in the society see women it would be a great sin*, *so*, *we do not allow women go outside for treatment”*** (Age-42, Sex-male, Education-class 9, Occupation-Imam).

For the same reasons as those mentioned above, four stakeholders and one TBA said that mothers prefer TBA for delivery because TBAs maintain veil (*purdah*). A female medical officer quoted

***“The community people think that*, *delivery at outside home is not regardful to them and they think it violates their purdah*. *Even at the time of delivery the presence of women’s mothers and her sister are not allowed and which also hampers purdah*. *Besides*, *if a woman has to go outside home for delivery*, *community people think that these are causes of sin with that family”*** (Age-29, Sex-female, Occupation-Medical Officer).

But in terms of religious views, some stakeholders and most of the village doctors reported some religious bigotry of Muslim people held about why they prefer home delivery but did not find this in the same way in the views of the Hindu participants. Both Muslim and Hindu participants talked about the religious fallacy found especially among the Muslim community.

#### Limited access of women to decision making in the family

Most of the women we interviewed opined that in case of their last delivery, the location of delivery and provider conducting the delivery was decided by the male and elderly members of the family. In this regard a woman said that,

**“*Conducting delivery at home is better for me*. *All of the family members take the decision and whatever they decide is better for me”*** (Age-22, Education-Class 5, Occupation-Housewife).

Another woman said that,

*“****We are house-wives; we always stay in house*, *we don’t have scope to move outside home and we don’t know about the outside world*, *how we can make the decision”*** (Age-23, Education-Class 5, Occupation-Housewife).

Even despite having desire, women do not have access to decision making. In this context, a respondent quoted

**“*I wished to have delivery at hospital but my family did not allow me to go to hospital”*** (Age-20, Education-Class 5, Occupation-Housewife).

#### Poor road conditions and lack of available transports

As previously mentioned, this study was conducted in a remote and hard to reach area of Bangladesh which is only accessible by boat during the rainy season. A few stakeholders reported that roads remain in poor conditions in all seasons. During the rainy season, boat is the only means of transportation; and even with a boat, it takes a long time to reach the hospital. On the other hand, during dry season motorbike is the sole mode of transportation which pregnant women cannot use. All of these transportation challenges are important reasons for home delivery by TBA in this particular area.

#### Fear regarding Caesarean delivery

Few of the study participants including women and TBAs expressed that if people go to private clinics or hospitals, the doctor will conduct a caesarean delivery instead of trying for a normal vaginal delivery. They also mentioned that caesarean delivery would cost a lot of money and the surgery might result in physical harm to the women. Based on these fears, many women prefer and opt for a home delivery.

Findings from our study reveal a both similar and dissimilar opinion about women’s birthing preferences among influential persons, community women, formal and informal providers, religious leaders, etc. All the groups of respondents uniformly cited the influence of poverty and tradition on women's preference for home delivery with the help of TBA. Both women and influential persons mentioned shyness to receiving treatment from male doctors and poor road conditions combined with a lack of available transportation as deterrents to facility deliveries. Meanwhile, only influential persons and informal providers mentioned religious beliefs as an influencing factor in the choice of home deliveries with TBA. Illiteracy and lack of knowledge about modern medical treatment was mentioned by only influential persons and formal providers as a reason for women's preference for home deliveries. On the other hand, only women discussed their limited access to decision making in the family and fear regarding caesarean delivery as reasons for their preference of delivering in the home; none of the other respondents mention these reasons.

While there was no marked difference in responses regarding the influence of traditional views, poverty, and lack of knowledge between Muslim and Hindu respondents, there was a difference in respondents' practices. Out of twelve women that we interviewed, seven were Muslim and five were Hindu. All of the Muslim respondents chose to give birth at home with a TBA while three Hindu women out of five opted for facility delivery with the help of qualified providers.

## Discussion

The wide range of study participants (e.g., formal and informal providers, women and stakeholders) mentioned several similar reasons why women prefer home delivery with a TBA. These include; poverty, strong faith in the skills and expertise of TBAs, prevailing traditional views and religious beliefs, poor road communication and transportation system in the area, lack of knowledge, fear regarding Caesarean delivery, and lack of female doctors in health facilities. Our study findings suggest that traditional views are the major reasons for giving birth at home with the help of TBAs. This correlates with other study findings in the region. For example, a study conducted in rural Punjab in India showed that the most common reasons for home delivery is traditional attitude [13]. Another study conducted in Pakistan by Shah N *et*.*al* (2010) showed that the most frequent reason for preferring home delivery is family tradition [14].

According to our study results, people consider TBAs as trusted and experienced figures in the community. This faith in TBAs usually prompts women to opt for home delivery with the help of TBA. Some studies show similar findings of heavy reliance on TBA for home delivery in rural settings. For example, Shah N e*t*.*al* (2010) study showed that people in Pakistan have strong faith -in Dai (TBA) based on family tradition [14]. Imagie A O, *et*.*al* (2002) conducted research in rural settings of Nigeria and found similar results of reliance on home delivery by the family 'Dai' (TBA) who is a well-known and trusted figure for the family [15].

Our study suggests that having a familiar figure like a TBA and known surroundings provide women a supportive environment in which to give birth. This is supported by Shiferaw *et*.*al* (2013) study in Ethiopia which showed that people think delivery should be conducted in a comfortable and supportive environment with the help of TBA that encourages home delivery [16].

Our study participants said that poor people and even those falling in the middle-income bracket cannot afford the cost of delivery at a private clinic or even at a government hospital. This leaves women with no choice but to deliver at home with a TBA. Other studies have shown that poverty is a major influence on the preference for home delivery and the use of TBA for deliveries. Poor people do not have sufficient money to spend on delivery and the cost of facility delivery is much higher than a home delivery by a TBA because a facility-based delivery requires patients to pay for the cost of medicines, hospital facilities, transport, etc. [14, 17]. Another study in rural Burkina Faso conducted by Some TD *et*.*al* (2011) documented that lack of money was a barrier for use of health facility for delivery. Due to this lack of money, many poor women had no other option than to deliver at home with the expectation that all would go well [18]. Titaley *et*.*al* (2010) also showed that financial limitations were a major constraint that prevented community members from accessing and using trained attendants and institutional deliveries [19].

According to the responses of our study participants, TBAs have a lot of experience, they are always available at the time of need and services may be obtained free of cost. A study conducted in rural Cambodia also found that TBA services can be obtained without spending cash money [7]. Sychareun V *et*.*al* (2012) showed how good experiences of giving birth at home influence other pregnant mothers to have delivery at home [20]. Our study also shows that community people prefer home delivery by TBA because of their longtime good experience generation to generation.

Our study shows that there is a widely held religious view that if pregnant women physically reveal themselves to male doctors, it is considered a religious sin as it is a gross violation of veil (*purdah*). Women prefer TBA for delivering at home since it allows this veil *(purdha*) to be strictly maintained. People also believe that their social status and prestige will diminish if delivery occurs outside the home. Pregnant women feel ashamed to share their problems with male doctors and are afraid to conduct delivery at facility. A study conducted in two provinces of Lao People’s Democratic Republic by Sychareun V *et*.*al* (2012) showed similar findings that most of the women feel shy and embarrassed by having a male attendant in the health facility during delivery. This same study also shows that pregnant women dislike the lack of privacy and confidentiality at the health facility and being naked during delivery made hospital deliveries less appealing than home deliveries [20]. On the contrary, a study conducted in West Java province in Indonesia shows opposite results as it did not find any religious misinterpretation in the preference of home delivery by TBA. The study might not have explored the religious norms in this regard [19].

In addition, according to the stakeholders in our study, older people, particularly mother-in-laws, view giving birth at home as an age-old tradition and therefore place restrictions on pregnant women for going to hospital. This was also demonstrated by Sychareun V *et*.*al* (2012) study which showed that if a woman and her husband preferred to deliver at a health centre, the mother, mother-in-law, aunt or even neighbors advised the women to deliver at home based on their own past experience of childbirth [20].

This study found that lack of education is an important factor that contributes to home delivery with TBAs. Other studies also describe how educational status influences care seeking from hospitals [13, 21–22]. In particular, women with more education are more likely to go to a facility for child birth. These studies also reveal that illiteracy and unawareness regarding delivery complications contribute to women preferring delivery at home.

Another important reason for giving birth with a TBA identified in our study area is the dilapidated condition of the roads and limited transportation which physically prohibits accessing delivery services from a facility. The situation worsens during rainy season when a boat is the sole means of transport. Even in the dry season when motorbike is the only transport, pregnant women are not able to use it. The results of this study are similar to several other studies which show that lack of transportation and long distances to health facilities contribute to preference for home delivery [20, 23–24]. A qualitative study conducted by Sychareun V *et*.*al* (2012) have come up with similar findings where we find lack of transportation is an important factor that leads to having home delivery[20]. Carter A *et*.*al* (2010) also showed that far distance or unavailability of transportation is the main barrier to receiving skilled maternity care [23]. Mekonnen *et*.*al* (2012) demonstrated that woman preferred home delivery due to the lack of transportation system and a very long distance of the health centre from their home [24].

In Bangladesh, recent BDHS data shows that caesarean delivery rates have increased from 9 percent in 2007 to 23 percent in 2014 [3]. Respondents in our study expressed concern that they will undergo a caesarean delivery if they go to a facility for their delivery. This concern also contributes to women’s fear of going to a facility and their preference for home delivery instead. This finding is supported by Carter A *et*.*al* (2010) study in rural and urban areas of Kenya, which found that women who had undergone a caesarian section were afraid of having another and as a result, planned for their next birth to be at home [23].

Rajalakshmi TK (2012) suggests that pregnant women choose home delivery because there is nobody to look after their other children and do the household chores [17]. While our study did not find such findings, women described that they feel comfortable when their husbands and other family members are nearby to provide support while giving birth at home. But in the case of a facility delivery, husbands and relatives are not allowed into the labor room [20]. Furthermore, Sychareun *et*.*al* (2009) study highlighted women’s fears and concerns about the position in which they are compelled to give birth at a hospital. Women usually deliver in sitting position at home and this may not be allowed in a hospital setting. In addition to this concern, women also express fear of the possibility of unknown medical procedures and an extended stay in the hospital following delivery [25]. Some of these fears and uncertainties regarding facility deliveries were also found among our study participants.

Our study findings show that women have hardly any access to decision making related to care seeking during delivery in the family. The lack of a voice within the family may be attributed to lack of education among the women. A baseline survey conducted in Sunamganj (2014) showed similar low education rates, with only 2% women having passed class 10 [10]. The same survey further showed that in terms of maternal and child healthcare seeking from outside the home, the main decision maker was the husband (50%) followed by the husband and wife together (28%), while the remaining is made by other family members. These findings demonstrate that, women in this setting have least access to decision making regarding health care seeking in the family [10].

## Limitations of the study

There are several limitations to this study. Majority of the KII respondents were men and that may skew the findings through an over-representation of men’s voices and perspectives on the issues explored through the interviews. Understanding the regional dialects was a challenge for proper communication with the respondents although we tried to minimize this by recruiting staff who were familiar with the local dialects. The study also experienced time constraints as it was done as a formative research prior to the commencement of an intervention. Provided with more time, we could have increased the sample sizes and included focus group discussions for triangulation of results obtained through the KIIs and IDIs. We conducted this study in the most remote rural parts of Bangladesh that might influence the results and may not be similar to other rural parts of Bangladesh. Finally, such qualitative exploration with the small sample size in a specific area is not representative to the whole country.

## Conclusion

This study explored the factors that influence women’s preference to give birth at home with the help of a TBA rather than going to a facility for her delivery. The study identified a wide range of factors, including; traditional views, poverty, strong faith in TBA and her experience, illiteracy and lack of knowledge regarding maternal health services, prevailing religious beliefs, poor road conditions and lack of available transport, and the fear of undergoing a caesarean delivery at health facilities. The study findings suggest that programs can consider ensuring access to quality delivery care particularly in remote and hard to reach areas. Introducing community skilled birth attendants may be a good option to ensure safe normal delivery care at the community level. Our findings also highlight the importance of involving influential persons such as: religious leaders, teachers, local male and female leaders and husbands of women, etc. in community mobilization activities to improve the maternal health knowledge and practices. Programs should also focus on targeted education and awareness campaigns to improve the community’s understanding and knowledge about maternal delivery care. For example, targeted interventions to older family members will help to ensure they are aware of the importance of having a skilled attendant during delivery. Educating the community with accurate information about the reasons for and likelihood of undergoing a caesarian section delivery may help to reduce the fear of caesarian deliveries at facilities.

The factors identified in this study for the preference of TBA and home birth could help policy makers and program implementers to adopt socially and culturally appropriate community based interventions that can contribute to the reduction of maternal mortality and morbidity and increase maternal health service utilization in underperforming areas such as Sylhet. To improve skilled attendance at birth and to reduce the heavy reliance on TBAs, there is a critical need to design appropriate interventions that address traditional and cultural misconceptions related to delivery and address culturally- informed demand creation efforts for facility delivery.
